# Kaposi sarcoma in a patient treated with upadacitinib for rheumatoid arthritis

**DOI:** 10.1016/j.jdcr.2023.07.019

**Published:** 2023-07-27

**Authors:** Cynthia Fournier, Maxwell Benjamin Sauder, Zaid Saeed Kamil, Marcus Otho Butler

**Affiliations:** a Princess Margaret Cancer Center, Toronto, Ontario, Canada; b Division of Dematology, Department of Medicine, University of Toronto, Toronto, Ontario, Canada; c Toronto General Hospital, Toronto, Ontario, Canada; d Department of Laboratory Medicine & Pathobiology, Anatomic Pathology, University of Toronto, Toronto, Ontario, Canada; e Division of Medical Oncology, Department of Medicine, University of Toronto, Toronto, Ontario, Canada

**Keywords:** herpesvirus, immunosuppression, iatrogenic immunosuppression, JAK inhibitor, Kaposi, Kaposi sarcoma

## Introduction

Upadacitinib is a Janus kinase (JAK) inhibitor that inhibits JAK1. It is approved for rheumatoid arthritis, psoriatic arthritis, atopic dermatitis, and ulcerative colitis. Kaposi sarcoma (KS) is a neoplasm of lymphatic endothelium-derived cells infected by human herpesvirus 8 (HHV-8).^1^ KS is subdivided into the following 4 clinical variants: classic, African endemic, because of iatrogenic immunosuppression, and associated with AIDS.^1^ Many immunosuppressants have been reported to trigger KS. To our knowledge, we report the first case of KS induced by upadacitinib.

## Case report

**Fig 1:**
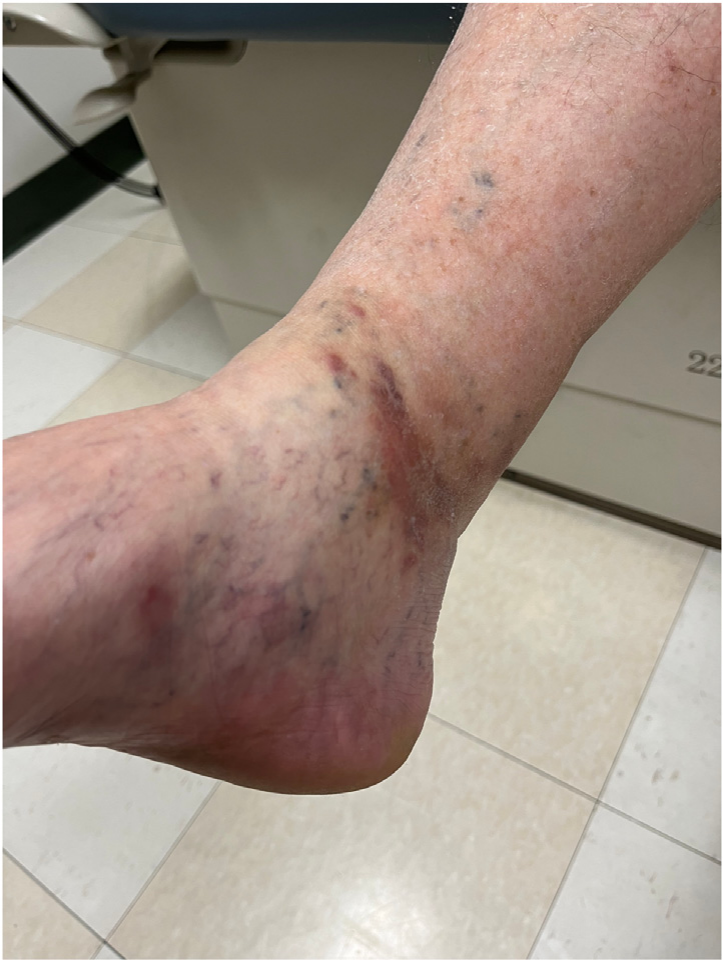
Violaceous papules and plaques on the distal aspect of the right leg with mild edema, consistent with Kaposi sarcoma.

**Fig 2:**
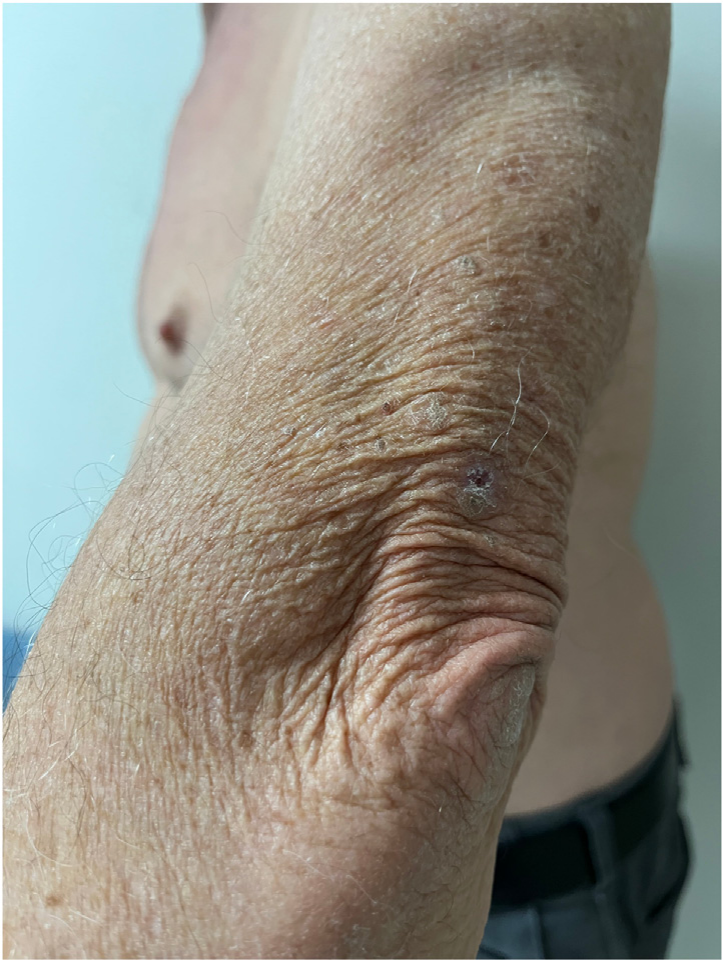
Crusted violaceous papule on the upper portion of the left arm, consistent with Kaposi sarcoma.

**Fig 3:**
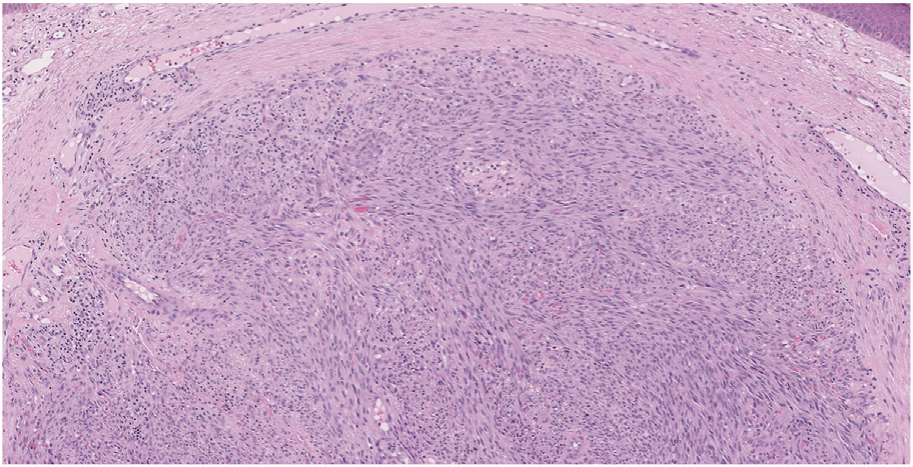
Intermediate power photomicrograph of Kaposi sarcoma showing a dermal-based, densely cellular, nodular spindle cell neoplasm composed of intersecting fascicles of atypical spindle cells with erythrocytes extravasation, ectatic vessels, scattered mitotic figures, and a mild lymphocytic infiltrate (hematoxylin and eosin stain).

**Fig 4:**
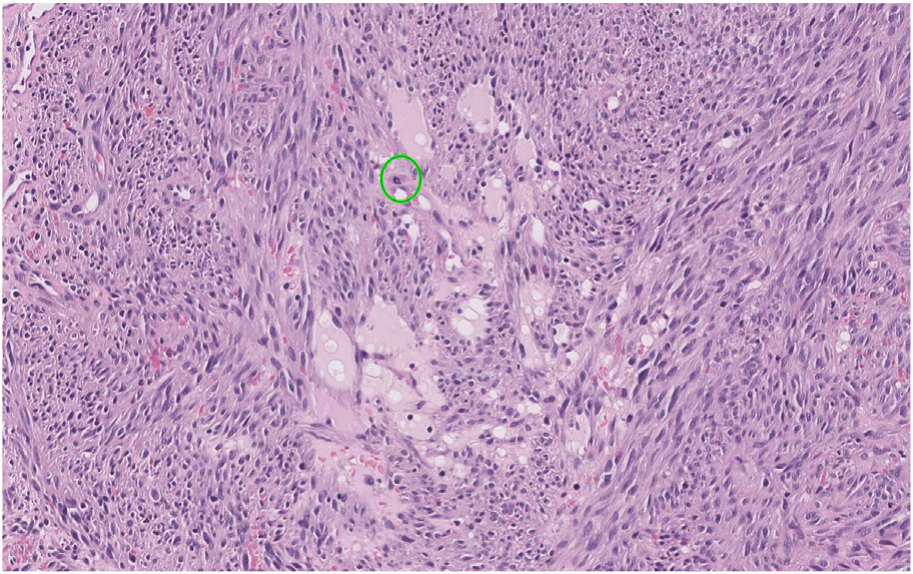
High-power photomicrograph showing a dermal-based, densely cellular, nodular spindle cell neoplasm composed of intersecting fascicles of atypical spindle cells with erythrocytes extravasation, ectatic vessels, and a mitotic figure (showed with a *green circle*) (hematoxylin and eosin stain).

**Fig 5:**
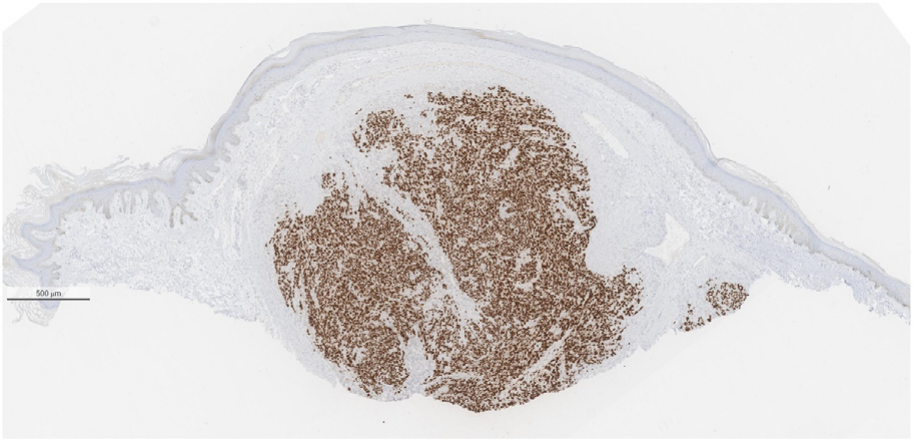
The neoplastic spindle cells are diffusely positive for human herpesvirus 8 (nuclear staining pattern).

**Fig 6:**
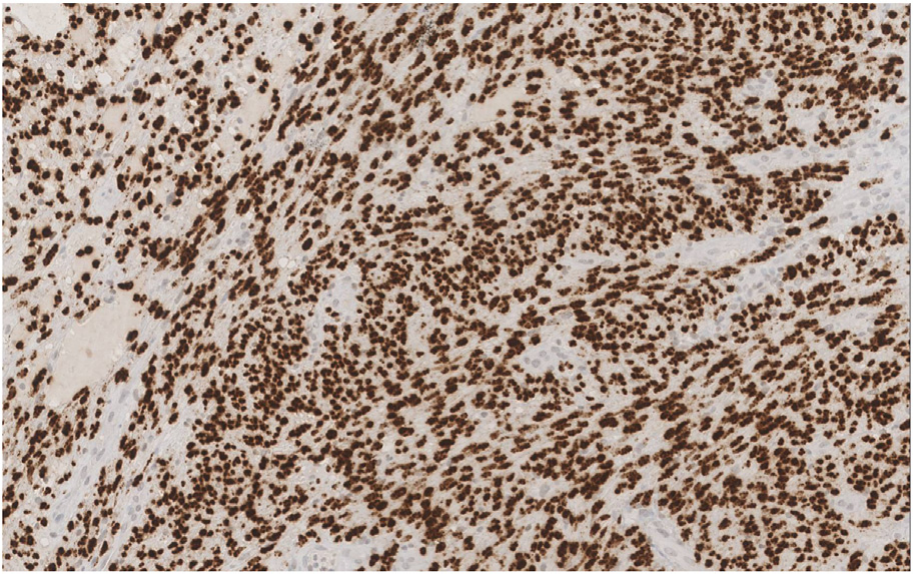
Human herpesvirus 8 showing nuclear staining of tumor cells. (Original magnification: 200×)

**Fig 7:**
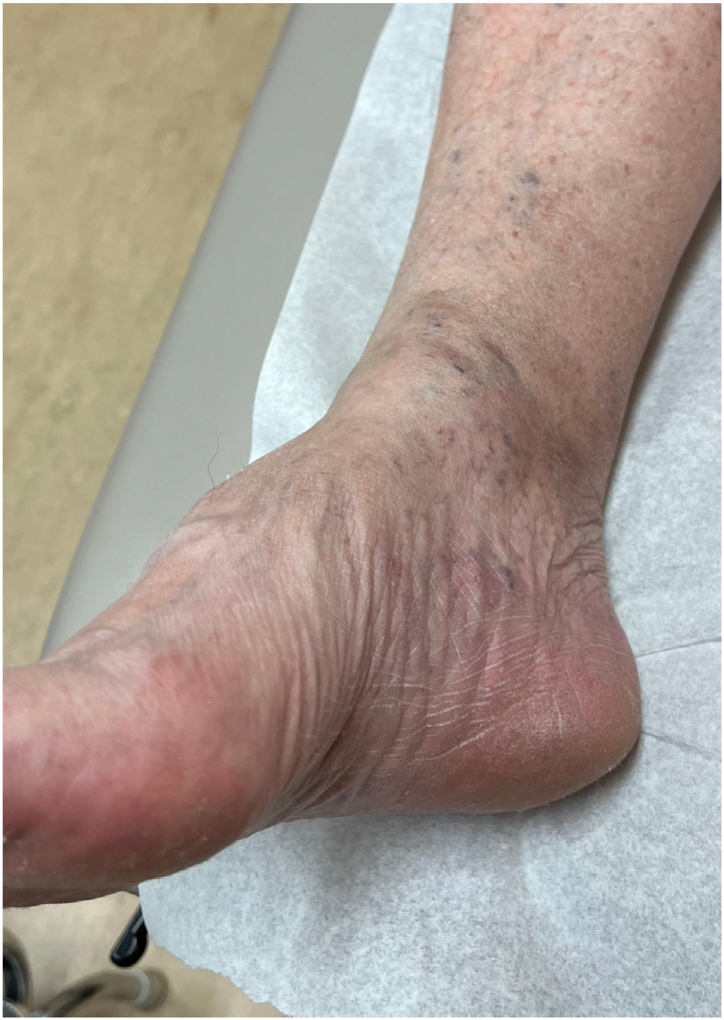
Postinflammatory hyperpigmented patches on the distal aspect of the right leg.

**Fig 8:**
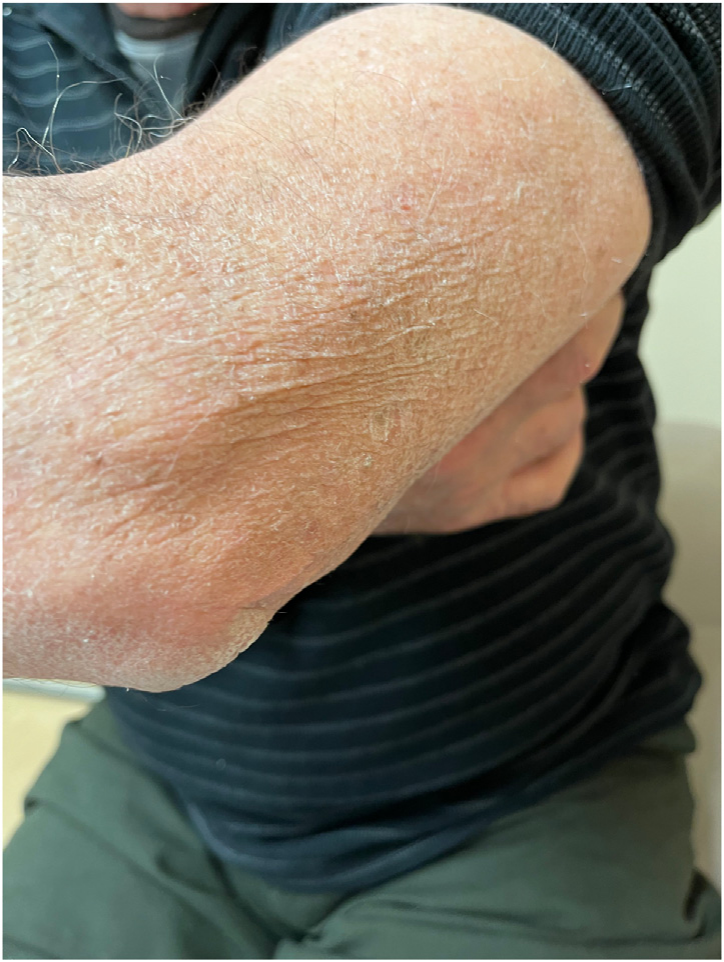
The violaceous lesion on the left arm disappeared 7 months after upadacitinib was discontinued.

A 77-year-old man presented to the medical oncology clinic with a new diagnosis of KS. He grew up in Italy and moved to Canada in his 20s. His medical history was remarkable for seronegative rheumatoid arthritis, dyslipidemia, and benign prostate hyperplasia. For his arthritis, the patient failed intra-articular steroid injections, low-dose methotrexate, and abatacept. He then started upadacitinib (15 mg daily). On upadacitinib, his arthritis was well controlled. Six months after starting upadacitinib, he developed asymptomatic violaceous papules, patches, and plaques, first involving his right foot, and then progressively appearing on the left foot (Fig 1), ankles, and left arm (Fig 2), with associated mild edema on the lower portion of the leg. Skin biopsies were conducted on the left arm and right plantar foot, and histopathology showed a dermal-based nodular spindle cell neoplasm composed of intersecting fascicles of atypical spindle cells, slit-like spaces, and mitoses (Figs 3 and 4). Immunohistochemistry demonstrated neoplastic cells diffusely positive for HHV-8 (Figs 5 and 6) and ETS-related gene (ERG) (vascular marker). The patient was diagnosed with KS because of iatrogenic immunosuppression, a JAK inhibitor in this case. The HIV test was negative. Computed tomography scans of the chest, abdomen, and pelvis excluded visceral involvement. The patient stopped upadacitinib, and 1 month after, he noticed spontaneous and simultaneous regression of all the lesions. He remained on active surveillance without other KS treatment, including topicals. Seven months after discontinuing upadacitinib, he presented with only postinflammatory hyperpigmented patches (Figs 7 and 8). His pedal edema had resolved. A skin biopsy was conducted on the right ankle and showed pigment incontinence without evidence of atypical vascular proliferation. HHV-8 stain was negative. The patient was in clinical and histopathologic remission following the discontinuation of upadacitinib.

He experienced arthritis flare with debilitating joint pain 4 months after discontinuing upadacitnib. He was prescribed hydroxychloroquine, sulfasalazine, and low-dose prednisone (10 mg daily for 1 month and then 5 mg daily). Medical oncology and rheumatology specialists decided that strong immunosuppressants and biologics should be avoided because of the theoretical risk of KS recurrence triggered by these medications.

## Discussion

KS is a neoplasm of lymphatic endothelium-derived cells infected by HHV-8.^1^ KS is subdivided into the following 4 clinical variants: classic, African endemic, because of iatrogenic immunosuppression, and AIDS-related. ^1^ There is a fifth variant recently described as KS in HIV-negative men who have sex with men.^2^ KS because of iatrogenic immunosuppression is mostly seen in solid organ transplant recipients.^1^ Risk increases with a more aggressive immunosuppressive regimen.^1^ The first step in the management of this KS subtype is to taper down the immunosuppression level to the lowest possible.^1^

JAK inhibitors are small molecules targeting the JAK-STAT pathway. JAKs are a family of intracellular proteins and include 4 tyrosine kinase members named JAK1, JAK2, JAK3, and TyK2.^3^ Each JAK inhibitor has a different affinity and selectivity for each tyrosine kinase members JAK1, JAK2, JAK3, and TyK2. For example, upadacitinib has a greater inhibitory potency against JAK1.

JAK inhibitors have immunosuppressive properties and can induce opportunistic infections. Herpes zoster is the most common infectious complication and is because of reactivation of latent varicella zoster virus.^4^ JAK inhibitors are also associated with a higher risk of primary herpes simplex virus and herpes simplex virus reactivation.^4^ The increased risk of herpes zoster and herpes simplex, both Herpesviridae infection, have a similar underlying mechanism. JAK signaling plays a key role in immunity against viruses.^3^ The immune response to intracellular pathogens, such as viruses is directed mainly by T helper cell type 1 (Th1).^3^ Interferon gamma and interleukin 12 are responsible for Th1 polarization and are mediated by the JAK-STAT pathway.^3^ Blockage of the JAK-STAT pathway induces a loss of Th1-driven immune response against viruses. If we extrapolate the data on varicella zoster virus and herpes simplex virus to another herpesvirus, the loss of Th1-driven immunity induced by JAK inhibitors may theoretically lead to the viral replication of HHV-8 and the occurrence of KS. In vitro studies showed that JAK2/STAT3 pathway inhibition enhances HHV-8 replication through reduction in proinflammatory cytokines interleukin 6 and tumor necrosis factor.^5^ JAK inhibitors also impair natural killer cell function and CD4-lymphocytes, which could also explain the increased risk of viral infections, including HHV-8. AIDS-associated KS occurs mostly in patients with low-CD4^+^ counts, so the impairment of CD4^+^ lymphocytes induced by JAK inhibitors could be another explanation for iatrogenic KS. The spontaneous resolution of KS after stopping the JAK inhibitor is in favor of a causative association between JAK inhibitors and KS.

There are 6 reported cases of iatrogenic KS induced by JAK inhibitors.^5^, ^6^, ^7^, ^8^, ^9^, ^10^ Four patients were treated with ruxolitinib,^5^, ^6^, ^7^, ^8^ 1 with baricitinib,^9^ and 1 with tofacitinib.^10^ The main characteristics of patients are presented in Table I.^5^, ^6^, ^7^, ^8^, ^9^, ^10^

**Table I:** Characteristics of the described case and 6 published Kaposi sarcoma cases induced by JAK inhibitors

References	Sex	Age, y	Country of birth	Disease treated with JAK inhibitors	JAK inhibitor	Time from JAK inhibitor initiation to KS diagnosis	HHV-8 serology status	Therapeutic management of KS	Outcome
Case described	M	77	Italy	Seronegative rheumatoid arthritis	Upadacitinib	6 mo	Unknown	Upadacitnib discontinuation	KS complete remission
Omine et al^6^	M	81	Japan (Okinawa islands)	Myelofibrosis	Ruxolitinib	6 mo	Unknown	Ruxolitinib discontinuation	Death 4 months after ruxolitinib discontinuation due to myelofibrosis progression
Moutel et al^7^	M	71	Italy	Myelofibrosis	Ruxolitinib	4 y	Serology positive, viral load 2799 copies/mL	Ruxolitinib discontinuation + doxorubicin	Death 2 weeks after ruxolitinib discontinuation due to KS deterioration
Loscocco et al^8^	M	56	Unknown, but he was treated in Italy	Essential thrombocytopenia	Ruxolitinib	7 y	Serology positive	Ruxolitinib discontinuation	KS complete remission
Tourlaki et al^5^	M	70	Italy	Myelofibrosis	Ruxolitinib	9 mo	Unknown	Ruxolitinib discontinuation + surgical excision + intralesional vincristine	KS complete remission
Martínez Pallás et al^9^	F	75	Unknown	Seronegative rheumatoid arthritis	Baricitinib	8 mo	Unknown	Baricitinib discontinuation + cryotherapy + topical imiquimod 5%	Good local control
Wetwittayakhlang et al^10^	M	61	Canada	Ulcerative colitis	Tofacitinib	2 y	Unknown	Tofacitinib discontinuation	KS complete regression

*HHV-8*, Human herpesvirus 8.

To our knowledge, we report the first KS case triggered by upadacitinib. Like 2 other published cases,^8^^,^^10^ our patient had KS remission after discontinuing the JAK inhibitor without the need for other KS-specific treatments. The patient remains at risk of KS recurrence should he receive another immunosuppressive treatment for his arthritis. He is also of Italian descent, and he could develop classic KS without the iatrogenic immunosuppression trigger. However, we believe that he was diagnosed with KS because of iatrogenic immunosuppression because it first clinically appeared after starting upadacitinib and it resolved shortly after its discontinuation.

The use of JAK inhibitors has significantly increased over the past years. We may expect to see more patients experiencing KS induced by JAK inhibitors in dermatology and medical oncology. Further studies may be required to better evaluate the risk of KS in patients receiving JAK inhibitors.

## Conflicts of interest

Dr Sauder did trials and was a consultant for AbbVie. Drs Fournier, Butler, and Kamil have no conflicts of interest to declare.
